# Transperitoneal laparoscopic pyeloplasty in children: does upper urinary tract anomalies affect surgical outcomes?

**DOI:** 10.1590/S1677-5538.IBJU.2017.0224

**Published:** 2018

**Authors:** João Arthur Brunhara, Paulo Renato Marcelo Moscardi, Marcos Figueiredo Mello, Hiury Silva Andrade, Paulo Afonso de Carvalho, Bruno Nicolino Cezarino, Francisco Tibor Dénes, Roberto Iglesias Lopes

**Affiliations:** 1 Unidade de Urologia Pediátrica, Faculdade de Medicina da Universidade de São Paulo - São Paulo, SP, Brasil

**Keywords:** Laparoscopy, Urinary Tract, Surgical Procedures, Operative

## Abstract

**Objective:**

To assess the feasibility and outcomes of laparoscopic pyeloplasty in children with complex ureteropelvic junction obstruction (UPJO) and compare to children with iso-lated UPJO without associated urinary tract abnormalities.

**Material and Methods:**

Medical records of 82 consecutive children submitted to transperi-toneal laparoscopic pyeloplasty in a 12-year period were reviewed. Eleven cases were con-sidered complex, consisting of atypical anatomy including horseshoe kidneys in 6 patients, pelvic kidneys in 3 patients, and a duplex collecting system in 2 patients. Patients were di-vided into 2 groups: normal anatomy (group 1) and complex cases (group 2). Demographics, perioperative data, outcomes and complications were recorded and analyzed.

**Results:**

Mean age was 8.9 years (0.5-17.9) for group 1 and 5.9 years (0.5-17.2) for group 2, p=0.08. The median operative time was 200 minutes (180-230) for group 1 and 203 minutes (120-300) for group 2, p=0.15. Major complications (Clavien ≥3) were 4 (5.6%) in group 1 and 1 (6.3%) in group 2, p=0.52. No deaths or early postoperative complications such as: urinoma or urinary leakage or bleeding, occurred. The success rate for radiologic improvement and flank pain improvement was comparable between the two groups. Re-garding hydronephrosis, significant improvement was present in 62 patients (93.4%) of group 1 and 10 cases (90.9%) of group 2, p=0.99. The median hospital stay was 4 days (IQR 3-4) for group 1 and 4.8 days (IQR 3-6) for group 2, p=0.27.

**Conclusions:**

Transperitoneal laparoscopic pyeloplasty is feasible and effective for the management of UPJO associated with renal or urinary tract anomalies.

## INTRODUCTION

Laparoscopic treatment of patients with ureteropelvic junction obstruction (UPJO) gained popularity in the last 20 years after the first cases were reported in the literature in 1993 for adults (1, 2) and in 1995 for children (3). However, although providing similar functional outcomes compared to open surgery (1, 4), and bringing the advantages of minimally invasive procedures (5), this approach requires advanced intra-corporeal operative skills and a longer learning curve when compared to open surgery (6).

Moreover, UPJO associated with complex anatomical anomalies such as horseshoe kidney (HSK), pelvic kidney (PK) and a duplex collecting system poses an additional challenge for the laparoscopic approach. Results of laparoscopic pyeloplasty in these settings are limited in the literature and data derive from small case series (7-9).

The aim of our study was to assess the feasibility and surgical outcomes of transabdominal laparoscopic pyeloplasty in children with complex UPJO compared to children with isolated UPJO (without associated urinary tract abnormalities).

## MATERIAL AND METHODS

A retrospective review of all pediatric laparoscopic pyeloplasties performed at our institution from November 2004 to November 2015 was performed. Data were retrieved from electronic and paper patient charts and operative reports. Institutional Review Board was granted and all data was handled by staff physicians with patient care responsibilities.

Between 2004 and 2016, 82 consecutive children were diagnosed with UPJO and submitted to transperitoneal laparoscopic pyeloplasty, medical records of these children were reviewed. The following surgical indications were considered for patients with UPJO: signs of kidney damage including impaired differential renal function <40% and/or features indicating poor drainage function like T1/2 >20 minutes after the administration of furosemide, and a plateau or ascending pattern of the excretion curve (10); associated symptoms and complications related to UPJO like ipsilateral flank pain, lithiasis, hypertension and hematuria. Eleven cases were considered complex, consisting of atypical anatomy including HSK in 6 patients, PK in 3 patients, and a duplex collecting system in 2 patients. Patients were divided into 2 groups: normal anatomy (group 1) and complex cases (group 2).

Demographics and clinical data evaluated included age, gender, laterality and presentation. Perioperative and surgical outcomes included operative time, surgical complications, length of hospital stay and symptoms, renal function and hydronephrosis improvement. Surgical complications were classified according to the Clavien-Dindo classification (11) adapted to children.

Postoperative renal evaluation was performed with ultrasound, diuretic renogram with diethylenetriaminepentaacetic acid (DTPA) or both at the discretion of the attending physician. Hydronephrosis on ultrasound was graded according to the Society for Fetal Urology (SFU) Grading scale (11). Ultrasonographic improvement was accepted as a decrease of at least one SFU grade and/or reduction of at least 7 millimeters in the anteroposterior diameter of the renal pelvis. DTPA excretory curve and half-time clearance improvement were analyzed for patients with pre and postoperative renograms (12). Improvement was considered as a change of pattern to a non-obstructive curve and/or a DTPA clearance half-time shorter than 20 minutes. Radiologic improvement was assessed either with ultrasonography and/or renogram. First post-op renal US was performed after 2-3 months after removal of the stent or the nephrostomy tube, every 6 months in the first year and then annually. Renal scans (DTPA) were performed 6 months after surgery and then repeated at each surgeon discretion if necessary. We have considered success of the treatment after 6 months of surgery (after US; DTPA) with resolution of symptoms, and if the improvement was sustained in the following studies until the last follow-up of each patient.

### Surgical Technique

For the normal anatomy cases (group 1) and for the two cases of duplicated collecting system, an open access was established through the umbilicus; a 10-mm (5-mm in some cases) port was inserted and insufflation was maintained at 10-12 mmHg. Then two 5-mm (3-mm in some cases) trocars were inserted under vision in the ipsilateral midclavicular line: one midway between the anterior superior iliac spine and the umbilicus, and the other two centimeters below the rib cage.

In HSK and PK cases, an open access was also established through the umbilicus with 5 or 10-mm port inserted and insufflation maintained at 10-12 mmHg. Additional 2 ports of 3-5-mm were used triangulated based on the position of the ureteropelvic junction (usually more medial and caudally located). The decision for port size selection in both groups was made at each surgeon discretion and was based mainly on patient age and weight.

The method of resection and re-anastomosis (dismembered) of the UPJ was performed as previously described by Anderson and Hynes in their original publication (13) through the laparoscopy approach. A transperitoneal approach was performed in all patients. The UPJO anastomosis was done in all cases with a 5.0 multifilament polyglactin absorbable with continuous running suture and a trans-anastomotic stent. A double-J stent (4 to 6 Fr) was placed in an anterograde fashion right after the posterior suture line was fashioned. In four cases, the stent was not placed in a antegrade manner due to not passage through the uretero-vesical junction. Of these, 3 patients belonged to group 1: two required a retrograde double-J placement and in one a nephrostomy tube was inserted. One case with a HSK (group 2) had a nephrostomy tube performed (6Fr) for urine drainage. Nephrostomy tube in these cases were fashioned with insertion of a multi-fenestrated feeding tube, 1.5cm above the suture line fixated by a 4.0 chromic purse suture and exteriorized through the flank. The catheter usually remained 5-7 days after surgery and was removed in the clinic when no urine output through it was observed.

A Foley catheter was left indwelling for 24-hours in all cases. Prophylactic antibiotics were administered by a single preoperative dose of 50 mg/kg cefazolin. Trimethoprim or cephalexin was administered until stent removal (two to four weeks after the procedure).

## RESULTS

For the analysis in this cohort, we considered only primary laparoscopic pyeloplasties. There were no significant differences in gender and age between groups. Overall, predominant gender was male 62% (44/71) of group 1 and 73% (8/11) of group 2, p=0.73. Laparoscopic pyeloplasty was performed with a mean age of 8.9 years (range 0.5 to 17.9 years) for group 1 and 5.9 years (range 0.5 to 17.2y) for group 2, p=0.08. Mean weight in group 1 was 35.7Kg (range 9-92Kg) and in group 2 was 27.3 (range 12.2-55Kg), p=0.18 (Table-1A).

**Table 1A:** Baseline: Demographic data.

		Group 1 (normal) n=71	Group 2 (anomalous) n=11	p value
**Age (years)**				
	Mean	8.9 (±5.2)	5.9 (±5.6)	0.08
**Weight (Kg)**				
	Mean	35.7 (±19.9)	27.3 (±13.3)	0.18
**Sex (%)**				
	Male	44 (62)	8 (73)	0.73
	Female	27 (38)	3 (27)	

Laterality and presentation are shown in Table-1B. In group 1, 20 cases (28.2%) were right sided and 51 (71.8%) were left sided. In group 2, 3 (27%) were right sided and 8 (73%) were left sided, p=0.99. No bilateral cases were present in this series. Preoperative flank pain was more common in the regular anatomy group with 40 patients (56.3%) presenting with this symptom, compared to only 3 cases in the complex group (27.2%), p=0.27. Forty-five percent of the patients in the complex group presented with prenatally diagnosed hydronephrosis which was higher than the normal anatomy group (28.2%) (Table-1B).

**Table 1B:** Clinical data.

		Group 1 (normal)	Group 2 (anomalous)	p value
**Laterality (%)**				
	Right Side	20 (28.2)	3 (27)	0.99
	Left Side	51 (71.8)	8 (73)	
**Presentation (%)**				
	Symptomatic	53 (74.6)	6 (55)	0.27
	Asymptomatic	18 (25.4)	5 (45)	

The median operative (skin incision to closure) was 200 minutes (IQR 180-230) for group 1 and 203 minutes (range 120-300) for group 2, p=0.15 (Table-1C). Blood transfusion and conversion to open surgery was not required.

**Table 1C:** Operative Time, crossing vessels, hospital stay, complications, clinical and radiologic improvement.

		Group 1 (normal)	Group 2 (anomalous)	p value
**Operative Time (min)**				
	Median (IQR)	200 (180-230)	203 (120-300)	0.15
**Crossing Vessels (%)**				
	Cases	30 (42.3%)	3 (27.2%)	0.46
**Hospital Stay (days)**				
	Median (IQR)	4 (3-4)	4 (3-6)	0.27
**Major Complications (%)**				
	Clavien ≥III	4 (5.6)	1 (6.3)	0.52
**Radiologic Improvement (%)**				
	Present	62/67 (92.5)	10/11 (90.9)	0.99
**DTPA improvement (%)**				
	Present	48/54 (88.9)	8/11 (72.7)	0.17
**Symptoms Improvement (%)**				
	Present	58/71 (82.1)	10/11 (90.9)	0.67

The surgical findings included crossing vessels in 30 patients (42.3%) for group 1 and 3 children for group 2 (27.2%), p=0.46. None in both groups required conversion to open surgery because of technical difficulties. In the complex anatomy group, crossing vessels were found only in horseshoe kidneys (50%; 3/6 patients). No crossing vessels were observed for pelvic or duplex kidneys.

Total complications (Clavien ≥1) were 14 (19.7%) for group 1 and 4 (36.3%) in group 2, p=0.71. Major complications (Clavien ≥3) were 4 (5.6%) in group 1 and 1 (6.3%) in group 2, p=0.52. The major complications on group 1 were: 2 obstructions right after the removal of the double-J, needing insertion of another stent and re-pyeloplasty posteriorly; 1 stent that migrated on the 10^th^ PO day and had to be exchanged; 1 case of a nephrostomy tube with impaired drainage of urine requiring a double-J catheter on the 7^th^ day after surgery. On the group 2 a patient with horseshoe kidney had a pyelonephritis 5 days after surgery with a mal-functioning nephrostomy tube. After IV hydration and introduction of antibiotics, a double-J catheter was inserted, and the nephrostomy tube removed. No deaths or early postoperative complications such as: urinoma or urinary leakage, bleeding, omental hernia occurred (Table-1C).

The success rate for radiologic improvement and flank pain improvement was comparable between the two groups. Radiological improvement (either seen at the renal scan and/or US), was present in 62 (92.5%) of valid cases in group 1 and 10 (90.9%) in group 2, p=0.99. For flank pain, success rate was 97.5% for primary and 100% for complex cases. The median hospital stay was 4 days (IQR 3-4) for group 1 and 4.8 days (IQR 3-6) for group 2, p=0.27 (Table-1C).

**Figure 1:**
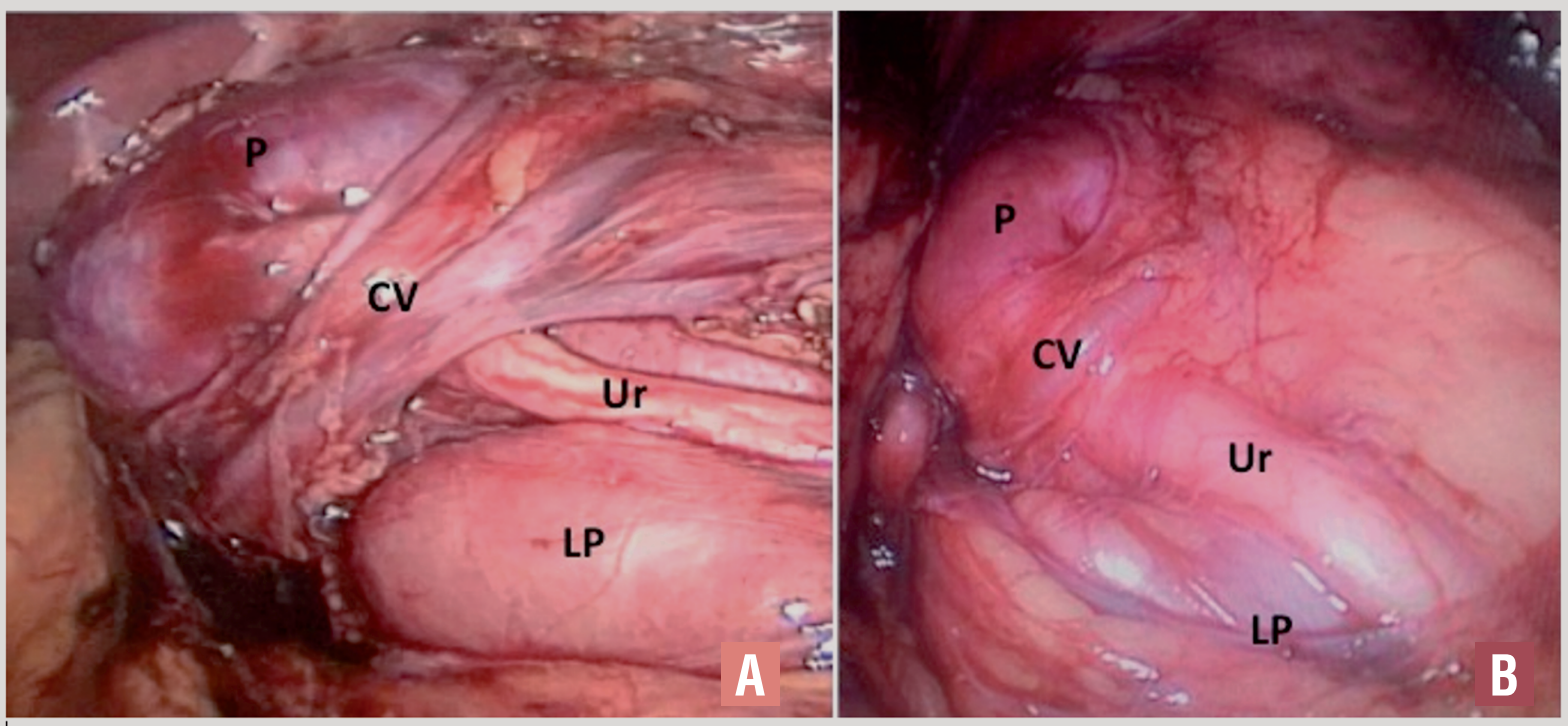
Horseshoe kidney in a six-year-old boy (A) and in a four-year-old boy (B). Presentation of crossing vessels **(CV)** on the anterior aspect of proximal ureter **(Ur)**; Renal Pelvis **(P)**; Lower Pole **(LP)**, of the kidney **(Isthmus)**.

**Figure 2:**
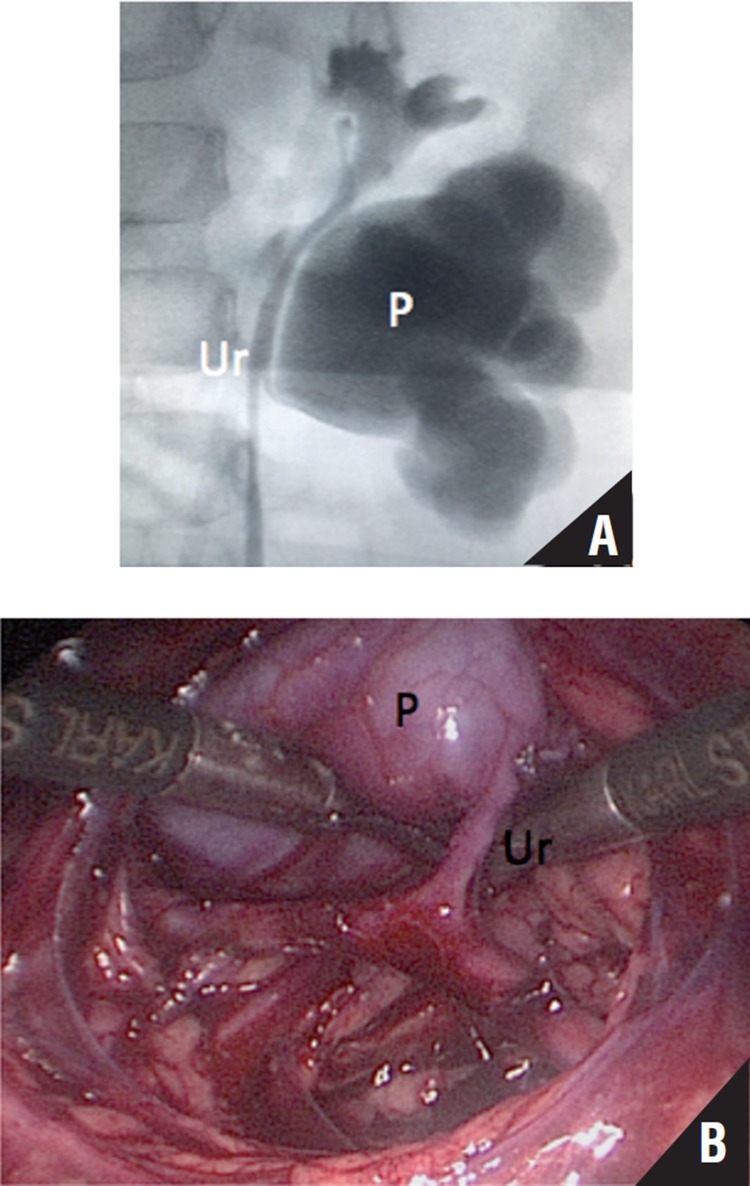
Duplicated collecting system in a nine-year-old girl. Pyelography **(A)**; Laparoscopic view **(B)**. Presentation of UPJO of the lower moiety; proximal ureter **(Ur)**; Renal Pelvis **(P)**.

**Figure 3:**
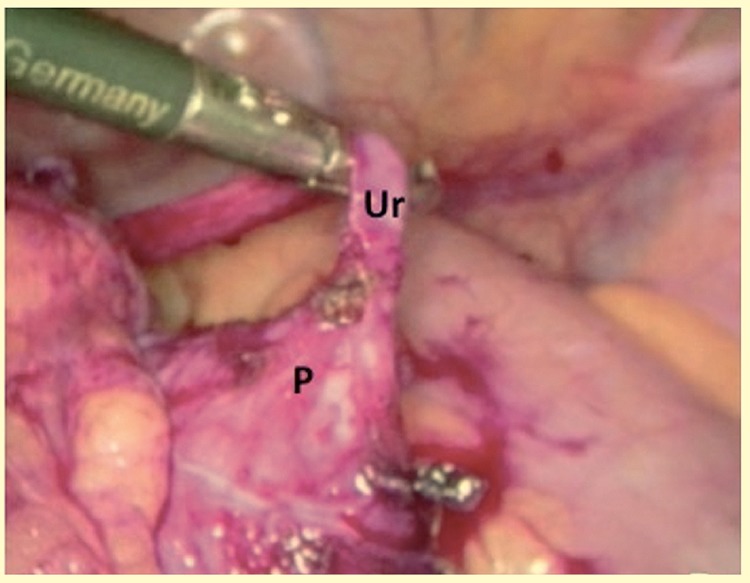
Transperitoneal aspect of a Pelvic Kidney in a one-year-old boy. Presentation of proximal ureter **(Ur)**; Renal pelvis **(P)**.

Regarding specifically the complex cases, more than half were symptomatic (6/11; 55%) and the others had hydronephrosis associated with worsening of pyelocalyceal dilatation on ultrasound and reduced renal function. UPJO associated with anatomic anomaly was diagnosed preoperatively (HSK in 6 patients, PK in 3 patients, and a duplex collecting system in 2 patients). Figures 1, 2 and 3 show examples of surgical findings in children with UPJO associated with horseshoe, duplex and pelvic kidney, respectively. For group 2, mean preoperative differential renal function, t1/2 and SFU grading hydronephrosis score were 37%, 40.9 min. and 3.4, respectively.

Mean surgical time was 203 min. (range 120-300). After a mean follow-up of 51 months (range 4-116), all children were asymptomatic and have improved the ultrasound parameters. All patients (except one) have improved the pattern of the DTPA curve to a non-obstructive pattern postoperatively (Supplementary Table-1).

**Supplementary Table-1:** 

Complex cases	Anomaly	Age at operation (mos)	Gender	Symptoms	Side	Preop SFU (US) Grade APD	Preop DTPA	OR time (min.)	Symptom Resolution	Postop SFU (US) Grade APD	Postop DTPA	Follow-up (mos)
1	Horseshoe Kidney	6	M	HDN antenatal	L	- IV - 35mm	- T>40min - IV	207	Yes	N/A	- T 23min - III	17
2	Duplex Kidney	55	F	HDN	R	- IV - 40mm	- T 28min - III	210	Yes	- II - 11mm	- T 21min - II	32
3	Pelvic Kidney	16	M	ITU	L	- III - 22mm	- T>40min - IV	300	Yes	- II - 15mm	- 6min - II	82
4	Horseshoe Kidney	55	M	HDN	L	- III - 18mm	- T>40min - III	180	Yes	- III - 15mm	- 5min - I	36
5	Pelvic Kidney	38	M	ITU	R	- IV - 85mm	- T>40min - IV	150	Yes	- III - 73mm	- T>40min - IV	116
6	Horseshoe Kidney	52	M	HDN	L	- IV - 50mm	- T>40min - IV	120	Yes	- II - 23mm	- T 32min - III	40
7	Horseshoe Kidney	207	M	Pain	L	- III - 34mm	- T 21min - III	200	Yes	- II - 24mm	- T 12min - II	78
8	Horseshoe Kidney	27	F	HDN	R	- IV - 48mm	- T>40min - IV	270	Yes	- III -10mm	- T 8 min - II	104
9	Horseshoe Kidney	84	M	UTI	L	- III - 29mm	- T>40min - IV	210	Yes	- II - 26mm	- T 15min - III	29
10	Pelvic Kidney	195	M	Pain	L	- III - 58mm	- T>40min - IV	150	Yes	- III - 42mm	- T 7min - II	28
11	Duplex Kidney	44	F	Pain	L	- II - 22mm	- T>40min - IV	240	Yes	- I - 9mm	N/A	4

**HDN** = Hydronephrosis; mos: months; **SFU** = Society of Fetal Urology; **US** = Ultrasonographic; **APD** = Anteroposterior diameter; **OR** = Operative; **N/A** = Not available

## DISCUSSION

Laparoscopic treatment for UPJO proved to be a safe and successful method, and has equal success rates in comparison to the open pyeloplasty with less morbidity (4). Rarely, UPJO might be associated with other congenital urinary tract anomalies such as a duplicated collecting system, HSK or PK which could require more wariness when performed laparoscopically. Previous studies in the adult population already showed good results in patients submitted to laparoscopic correction of UPJO associated with other kidney anomalies (14, 15). Here we present our experience, with the transperitoneal laparoscopic approach to treat all surgically indicated UPJO cases associated with the existence of an upper urinary tract anomaly in children. We believe that this kind of approach can bring us a several number of advantages such as: the inherent lower morbidity of a minimally invasive procedure; an excellent global vision of the atypical anatomy like aberrant vessels and abnormal ureteral position; and a comparable operative time with laparoscopic pyeloplasty in regular anatomy kidneys.

In our study, we presented the surgical treatment of UPJO in a variety of associated congenital anatomical anomalies. Horseshoe kidney is usually associated with some contributing factors to UPJO such as a high insertion of the ureter into the renal pelvis, abnormal ureteral course anterior to the isthmus and anomalous blood supply to the kidney (16, 17). Shadpour et al. showed a success rate of 93.3% (using DTPA exam) in the treatment of a cohort of fifteen patients with horseshoe kidney and they found eight kidneys (53.3%) with anterior crossing vessels (18). In parallel, we showed six cases with UPJO associated with horseshoe kidneys and found three out of six (50%) with the presence of a crossing vessel (Figure-1). The transperitoneal laparoscopic approach specifically for HSK can make the procedure easier after proper identification and careful dissection of possible anomalous vessels (Figure-1). The medially and anteriorly position of the UPJ typically facilitate the dissection and suture after these vessels are properly identified. The presence and position of these vessels might be prevented and anticipated by a MR urography pre-operatively (8). However, this could imply an additional general anesthesia to perform this image study in children.

Ureteral duplication with UPJO is a rare anomaly and surgical treatment for UPJO in duplex kidneys has rarely been reported in small series (9, 19, 20). It is important to study preoperatively and differ partial from complete duplex systems and to exclude coexistent urologic anomalies, especially vesicoureteral reflux and ureterocele. In the two cases reported in this paper, a retrograde pyelography was performed at the same time of the surgical intervention to confirm the diagnosis (Figure-2). In this kind of anomaly, the transabdominal technique has also been proved useful. After correct identification of the anatomy, the type of anastomosis can be decided based on the individualized characteristic of each case.

The incidence of UPJO in pelvic kidneys is relatively high and estimated at 22-37% (21). Pelvic kidneys have abnormal migration and rotation leading to abnormal vascularization and in some cases to high insertion of the ureter. Muller et al. published their experience in the treatment of 5 cases of UJPO in pelvic kidneys with laparoscopic approach with no morbidity and durable success rate (7) comparable with open approach series (22). In our study, we present three cases with complete resolution of symptoms with minimally invasive access (Figure-3).

The limitation of our study is that it is a retrospective case series. Furthermore, reconstructive laparoscopic procedures like pyeloplasties impose a steep learning curve that cannot be easily surpassed by low-volume centers specially when dealing with complex cases. This issue could be overcome with the use of robotic surgery, which allows more dexterity for complex and delicate sutures like the UPJ anastomosis. Nevertheless, due the high financial burden, unfortunately this is not a medical resource available for all, and our unit does not have access to robotic surgery.

However, our series along with the other small reported series showed that transperitoneal laparoscopic pyeloplasty is a safe procedure with good results, minimal morbidity and no long-term complications, even in cases of congenital associated anatomical abnormalities.

## CONCLUSIONS

Our data shows that in children with anomalous kidneys, transperitoneal laparoscopic pyeloplasty is a safe procedure with good results, minimal morbidity and no long-term complications. Surgical and clinical outcomes are no different from children with UPJO but without associated anomalies submitted to primary laparoscopic pyeloplasty.

## ABBREVIATIONS

UPJO: = Ureteropelvic junction obstruction
HSK: = Horseshoe kidney
PK: = Pelvic kidney
DTPA: = Diethylenetriaminepentaacetic acid
SFU: = Society for Fetal Urology
